# A237 ROTAVIRUS VACCINE SAFETY IN INFANTS EXPOSED TO BIOLOGICS IN UTERO IN AN INFLAMMATORY BOWEL DISEASE POPULATION: A PROSPECTIVE COHORT STUDY

**DOI:** 10.1093/jcag/gwad061.237

**Published:** 2024-02-14

**Authors:** Y Jeyakumar, Z Mohmand, K O'Connor, P Piche-Renaud, S Morris, V Huang

**Affiliations:** Internal Medicine, University of Toronto, Toronto, ON, Canada; Internal Medicine, University of Toronto, Toronto, ON, Canada; Sinai Health, Toronto, ON, Canada; The Hospital for Sick Children, Toronto, ON, Canada; The Hospital for Sick Children, Toronto, ON, Canada; Sinai Health, Toronto, ON, Canada

## Abstract

**Background:**

Biologics, the mainstay of therapy in patients with moderate-to-severe inflammatory bowel disease (IBD), undergo placental transfer during the second trimester of pregnancy and may remain detectable in the blood of exposed infants for up to 12 months. Due to concerns regarding the risk of immunosuppression and adverse events following immunization with live attenuated vaccines in infants exposed to biologics in utero, current guidelines recommend avoiding live vaccines in the first 6 to 12 months of life.

**Aims:**

This study investigates whether the live rotavirus vaccine can safely be administered in infants exposed to biologics in utero.

**Methods:**

We conducted a single-centre prospective cohort study, which included infants exposed to biologics in utero and born to mothers with IBD between October 1, 2020 and June 30, 2022. Infants were included in the study if they were assessed by Paediatric specialists at the Special Immunization Clinic (SIC) at SickKids Hospital (Toronto, Ontario). Mothers were followed by Gastroenterologists at the Special Pregnancy IBD Clinic at Mount Sinai Hospital (Toronto, Ontario). Data were collected on biologic agent exposure history, infant immune function testing, SIC recommendations for rotavirus vaccination, rotavirus vaccine series completion, and adverse events after immunization. Adverse events following immunization included fevers, vomiting, diarrhea, and/or intussusception within 42 days following immunization.

**Results:**

22 infants were included in the study. Of the 22 infants, 5 (22.7%) were exposed to infliximab in utero, 10 (45.5%) were exposed to adalimumab, 3 (13.6%) were exposed to vedolizumab, and 3 (13.6%) were exposed to ustekinumab. Of the 22 infants, 18 (81.8%) were recommended to receive the rotavirus vaccine by the SIC. 1 (0.05%) was not recommended to receive the vaccine due to neutropenia and low CD54 count on lymphocyte testing. The recommendation given to 3 infants is unknown. Of the 18 infants who were recommended to receive the vaccine, 16 (88.9%) received the vaccine and had no adverse events following immunization. 1 (6%) infant did not receive the rotavirus vaccine despite it being recommended due to parental choice. The rotavirus vaccination status and response in 1 infant recommended to receive the vaccine, is unknown.

**Conclusions:**

Administration of the rotavirus vaccine in biologic-exposed infants did not result in adverse events in our study cohort. Although guidelines currently advise against live vaccinations in infants exposed to biologics in utero, specialist assessment of infant immune function can help encourage safe live vaccination uptake.

**Funding Agencies:**

None

